# Hospital Resources for Pandemic Influenza

**DOI:** 10.3201/eid1403.071570

**Published:** 2008-03

**Authors:** Michael P. Dailey

**Affiliations:** * North Fulton Regional Hospital, Roswell, Georgia, USA

**Keywords:** Influenza, human, disease outbreaks, health resources, letter

## Abstract

Hospital Resources for Pandemic Influenza

**To the Editor:** In their November 2007 article, Pandemic Influenza and Hospital Resources, Nap et al. evaluated hospital resources for pandemic influenza in the northern part of the Netherlands (*1*). Their results can be compared with those that I have described for the combined suburban communities of Roswell and Alpharetta, Georgia, USA (*2*). The Netherlands evaluation assumed that antiviral drugs will be available and will reduce hospitalizations by 50% and deaths by 30%. In view of the uncertainty of effective antiviral drugs and timeliness of vaccines, I did not estimate their effects. Nevertheless, several issues warrant comparison.

The plan for the Netherlands has no provisions for urgent care, i.e., parenteral fluids or antimicrobial drugs that are administered to ambulatory patients who are not hospitalized. Nap et al. may not perceive a need for enough beds to handle surge capacity. Allowing for 30% of beds to be used for patients with conditions other than influenza, they report a maximum availability of 232 beds per 100,000 population for pandemic influenza patients, and they estimate use of 72 beds per 100,000 in the pandemic model. In contrast, a maximum of 47 beds per 100,000 are available in Roswell/Alpharetta. Availability of beds in intensive care units, however, is identical for both regions, at 8 beds per 100,000 population.

The Netherlands plan calls for intensified treatment evaluation in 48 hours to withdraw care from patients who have little chance for recovery. Because most patients can be expected to have pneumonia and 2-organ failure (on average), a 50% mortality rate can be expected. In US hospitals, withdrawing care is difficult, even if mortality rates are expected to be 75% or 90% during acute illness with organ failure.

The pandemic influenza resource evaluation from the northern part of the Netherlands provides a useful contrast with at least 1 US hospital. The dramatic difference in bed availability highlights the potential challenges involved in local planning. The surge capacity limits in Roswell/Alpharetta led us to consider an alternative infusion center to provide care during an influenza pandemic.
